# Oral microbiome of the inner surface of face masks and whole saliva during the COVID-19 pandemic

**DOI:** 10.3389/froh.2023.1178020

**Published:** 2023-07-14

**Authors:** Yeon-Hee Lee, Hyeongrok Kim, Dae Wook Heo, In-Suk Ahn, Hee-Kyung Park

**Affiliations:** ^1^ Department of Orofacial Pain and Oral Medicine, Kyung Hee University Dental Hospital, Kyung Hee University School of Dentistry, Seoul, Republic of Korea; ^2^ Life Sciences Lab, Denomics, Seoul, Republic of Korea; ^3^ Department of Oral Medicine and Oral Diagnosis, Dental Research Institute, Seoul National University School of Dentistry, Seoul, Republic of Korea

**Keywords:** mask, saliva, bacteria, microbiome, xerostomia, shannon diversity index

## Abstract

Wearing a face mask was strongly recommended during the COVID-19 pandemic. The purpose of this study was to investigate the diversity of the oral microbiome, the abundance of each bacterium on the inner surface of the mask, and the effects of xerostomia on the microbiota. The study was conducted on 55 generally healthy adults (45 women and 10 men, mean age 38.18 ± 12.49 years). Unstimulated flow rate (UFR) and stimulated flow rate (SFR) were measured in whole saliva samples collected for each condition. The 14 major oral bacterial species, including *Porphyromonas gingivalis (P. gingivalis)*, *Lactobacillus casei (L. casei)*, *Tannerella forsythia (T. forsythia)*, and *Treponema denticola (T. denticola)* on the inner surface of the mask and in the UFR and SFR samples, were analyzed by real-time PCR. We found that the total DNA copy number of oral bacteria was significantly higher in UFR and SFR than in the mask (*p *< 0.001). On the inner surface of the mask, *P. gingivalis* and *L. casei* were the most abundant Gram-negative and Gram-positive species, respectively*.* The oral microbiome profile of the mask differed from that of the UFR and SFR samples. Shannon's diversity index was also significantly higher in the UFR and SFR than in the mask (2.64 ± 0.78, 2.66 ± 0.76, and 1.26 ± 1.51, respectively, *p *< 0.001). Shannon's diversity index of UFR and SFR had a significant positive correlation with each other (*r* = 0.828, *p* < 0.001), but there was no significant relationship with Shannon's diversity index of mask. Red complex abundance, including *P. gingivalis, T. forsythia,* and *T. denticola*, was significantly higher in UFR than in the mask. Interestingly, the DNA copy number of each of the 14 bacteria, the total bacterial amount, and Shannon's diversity index did not differ in the absence or presence of xerostomia (*p* > 0.05). In summary, oral bacteria migrated to and existed on the inside of the mask, and the presence of xerostomia did not affect the bacterial profiles. The inner surface of the mask had an independent oral microbiome profile, although this showed lower quantity and diversity than the UFR and SFR samples.

## Introduction

Coronavirus disease 19 (COVID-19), which started in December 2019, was officially declared an infectious disease pandemic by the World Health Organization (WHO) on January 30, 2020. A total of 688 million confirmed cases and 6.87 million COVID-19 related deaths were reported by early May 2023 [WHO Coronavirus (COVID-19) Dashboard, https://covid19.who.int]. Wearing a facemask was mandatory worldwide during the COVID-19 pandemic; however, restrictions on mask-wearing in public places have now been lifted (1). Epidemiologists and virologists agree that COVID-19 mainly spreads from infected symptomatic individuals to others in close contact via respiratory droplets and aerosols, and the mouth and nose are the primary modes of transmission (2, 3). Guidelines for the general public from the Center for Disease Control, WHO recommend wearing facemasks to minimize the risk of transmission of COVID-19 (4). Using masks in combination with other precautions, such as vaccination, frequent hand washing, and physical distancing, can help reduce the spread of COVID-19 (5). Therefore, face masks are important to prevent the transmission of severe acute respiratory syndrome coronavirus type 2 (SARS-CoV-2) during periods of lack of therapeutic intervention.

Despite the obvious usefulness of the mask in preventing virus transmission (6), long-term use of masks can lead to several important health problems. First, this can increase physiological and psychological burdens and even reduce work efficiency and academic ability (7, 8). Long-term use of masks also causes physical side effects such as headaches and shortness of breath due to reduced oxygen supply (9). A tight-fitting mask disrupts thermal equilibrium and raises CO_2_ levels. CO_2_ is a well-known respiratory stimulant, and the accumulation of exhaled CO_2_ between the mask and face can lead to increased lung ventilation and respiratory activity, as well as changes in skin temperature and humidity (10). Additionally, CO_2_ accumulation in the chamber created by the mask can cause mental confusion and cognitive impairment. Skin-related side effects such as skin dryness, itching, rashes, and acne may also occur due to mask use (11). The increase in skin irritation is explained by blockage of the facial ducts due to compression in a humid environment under a tight-fitting mask (12). However, potential bacterial growth and their origins in the humid and warm environment caused by masks have not been studied.

The warm and humid environment of the oral cavity covered by masks is a unique habitat for oral bacteria. The oral cavity is coated with saliva and provides a diverse environmental habitat for more than 700 species of oral microbiota (13). Human saliva is composed of over 98% water, with the remainder consisting of various electrolytes, mucins, enzymes, nutrients, and antibacterial substances (14). Saliva also contains an oral microbiome and a mixture of bacterial communities (15). The hydration of the oral cavity and the composition of saliva therein may be important for the maintenance of oral microbiome symbiosis and occurrence of dysbiosis. To date, few significant studies on masks and the attendant microbiome have been published. A study conducted by Au et al. in 2022 reported that continuously wearing a mask for 2 months did not significantly change the salivary oral microbiome compared to those with minimal mask use (16). However, only the saliva microbiome but not the oral microbiome inside the mask was directly investigated, and the participants were limited to young dental students with an average age of 26.36 ± 1.58 years (16). Park et al. investigated bacteria and fungus obtained from masks by culturing samples on agar and counting the number and size of microbial colonies over time (17). They found that prolonged mask wearing did not alter the bacterial composition. However, objective quantitative and qualitative analyses of bacteria sampled from different age groups are required to draw definitive conclusions about these findings.

We hypothesized that the salivary microbiome could proliferate on the inner surface of the mask and aimed to investigate which specific bacterial cohorts proliferate inside the mask. In addition, we investigated the relationship between bacteria obtained from the inner surface of the mask, and those obtained from whole saliva under conditions of unstimulated flow rate (UFR) and stimulated flow rate (SFR). This study distinctively analyzed the bacteria in the saliva and masks of volunteers of various ages using the PCR technique. Furthermore, one critical complaint from people wearing a mask for prolonged periods is dry mouth. Xerostomia can be caused by decreased salivary flow rates and decreased water intake with prolonged mask use (18). The results of this study provide a rationale for using masks that are as clean as possible, a pathophysiological explanation for dermal consequences or skin problems caused by the salivary microbiome in the area covered by the mask, and evidence for frequent mask changes.

## Materials and methods

### Study population

A total of 55 healthy individuals (45 females and 10 males; mean age, 38.18 ± 12.49 years) participated voluntarily in this study at the Kyung Hee University Dental Hospital by advertising between September 1, 2021 and October 31, 2021. The research protocol for this study was reviewed for compliance with the Declaration of Helsinki and approved by the Institutional Review Board of Kyung Hee University Dental Hospital in Seoul, South Korea (KHD IRB, IRB No-KH-DT21023). Informed consent was obtained from all participants. We determined whether patients had subjective dry mouth or xerostomia, then grouped them into a group with xerostomia (14 total, 12 females; 36.43 ± 12.99 years) and without xerostomia (41 total, 33 females; 38.78 ± 12.42 years). They were also requested to complete questionnaires used for the analysis of gender, age, mask-wearing duration, salivary pH, salivary buffer capacity, and xerostomia. The oral tissues, including periodontal tissues, buccal mucosa, and general condition of all participants were determined for health status. Our study on halitosis using the same research methods has recently been published (19).

The inclusion criteria were as follows: medically healthy adults with healthy periodontal condition, having lost <2 teeth in the permanent dentition, able to voluntarily read and judge the consent form, and able to participate. Individuals taking drugs that affect salivation, including psychiatric medications such as anti-anxiety or sleeping pills and antibiotics, were excluded. Pregnant or lactating women and adults non-compliant with clinical examination and/or sample collection were also excluded. Furthermore, we excluded adults with systemic diseases or disabilities that influence the capacity for oral health self-care or cause salivary gland dysfunction, as well as adults with a partial denture or fixed orthodontic device(s) that can alter the oral microbiome and salivary flow rate. Finally, participants whose data collection was insufficient were excluded and those who dropped out or could no longer participate due to research-related circumstances were also excluded. Careful attention was paid during the entire protocol of obtaining saliva and mask samples and identifying oral microorganisms to prevent further contamination by the researcher. Researchers also wore a mask, thoroughly sanitized their hands, and wore disinfected dental gloves, which were replaced for each participant. For laboratory analysis, the experimental table was wiped with alcohol before the experiment. All items and reagents used in the experiment that had direct contact with the sample, such as pipette tips and tubes, were sterilized before use and discarded after a single use. A centrifuge with a lid was used to prevent contamination by aerosols. Reagent preparation and reactant preparation for the PCR reaction were performed on a clean bench.

### Collection of unstimulated and stimulated whole saliva

Before the saliva sampling session, participants were instructed to refrain from consuming caffeine and/or nicotine for at least 4 h and from consuming alcohol for at least 24 h. Whole saliva samples, both stimulated and unstimulated, were collected between 9:30 and 11:30 a.m. to minimize diurnal variability, and the mean time difference between waking up and collection was 3 h. All participants were instructed to abstain from eating, drinking, and brushing their teeth before saliva sample collection. Unstimulated whole saliva was collected for 10 min using the spitting method. Stimulated whole saliva was collected for the next 5 min while chewing 1 g of gum base, following a 2-min pre-stimulation period to remove saliva retained in the ducts. The UFR and SFR of saliva are expressed in mL/min.

### Salivary pH and salivary buffer capacity

To determine the salivary pH and buffer capacity, GC Saliva Check Buffer kits (GC, Tokyo, Japan) were used. After measuring the UFR, a salivary pH test strip was placed into the resting unstimulated whole saliva sample for 10 s. Subsequently, the color obtained was compared with the test chart included in the kit. A pH value of 6.8 or higher corresponds to healthy saliva, whereas a value lower than 6.6 is characterized as acidic. To measure the saliva buffering capacity, stimulated whole saliva was collected and used to dampen three areas of the test strip using a pipette. After 2 min, the colors appearing in the three areas were scored as follows: green, 4 points; green/blue, 3 points; blue, 2 points; red/blue, 1 point; and red, 0 points. The result was interpreted using the scheme in the kit, where each resulting total value corresponds to a degree from “very low” to “normal” salivary buffer capacity (minimum 0 points, maximum 12 points), as follows: 0–5: very low; 6–9: low; 10–12: normal.

### Identification and quantification of oral Bacteria

The amount of bacterial DNA, bacterial community composition, and individual abundance of oral bacterial species were determined.

#### Oral bacteria

In the UFR and SFR saliva samples, the absolute amount and abundance of fourteen anaerobes were identified: *Aggregatibacter actinomycetemcomitans (A. actinomycetemcomitans), Prevotella intermedia (P. intermedia), Prevotella nigrescens (P. nigrescens), Eikenella corrodens (E. corrodens), Campylobacter rectus (C. rectus), Fusobacterium nucleatum (F. nucleatum), Porphyromonas gingivalis (P. gingivalis), Treponema denticola (T. denticola), Tannerella forsythia (T. forsythia), Lactobacillus casei (L. casei), Streptococcus mutans (S. mutans), Streptococcus sobrinus (S. sobrinus), Parvimonas micra (P. micra)*, and *Eubacterium nodatum (E. nodatum)*. Bacteria inside the mask (mask sample) were collected by washing the inside of the KF94 mask worn by the participants for more than 3 h with 20 ml preservation solution. The KF94 mask effectively blocked approximately 94% of airborne fine particles, including viruses and bacteria.

#### Bacterial-DNA isolation

For DNA isolation, the mask, UFR, and SFR samples were vigorously vortexed. From each sample, 500 μl was pipetted and added to a tube containing 500 μl of lysis buffer (5 mM EDTA, 5 M guanidine hydrochloride, and 0.3 M sodium acetate). Samples were vortexed for mixing, and the tubes were incubated at 65°C for 10 min. S2 buffer (0.25 g/ml silicon dioxide; Merck KGaA, Darmstadt, Germany) was thoroughly mixed by vortexing. Thereafter, 20 μl of this buffer was added to the sample-lysis buffer mixture. After vortexing, the tubes were incubated for 5 min at room temperature with intermittent inversion by an automatic system. The mixture was centrifuged for 30 s at 5,000 rpm, and the supernatant was then carefully removed. One milliliter of PureLink (Invitrogen Corporation, Carlsbad, CA, USA) and PCR purification washing buffer 1 [50 mM 3-(N-morpholino) propane sulfonic acid buffer, pH 7.0, with 1 M sodium chloride] was activated by adding 160 ml of 100% ethanol. This solution was added to the tube and mixed by vortexing until the beads were completely resuspended. After one more centrifugation at 5,000 rpm for 30 s, the supernatant was carefully removed. Thereafter, 1,000 µl of wash buffer 2 (ethanol) was added and vortexed until the beads were completely resuspended. Finally, the tube was centrifuged again at 5,000 rpm for 30 s, and the supernatant was completely removed. The elution buffer of 100 μl (100 mM Tris-HCl, pH 7.5, and 1 M EDTA) was added to the tube and vortexed to resuspend the beads. The tubes were further incubated at 65°C for 10 min, and the DNA was isolated. For PCR analysis, samples were centrifuged at 13,000 rpm for 5 min, and the supernatant was transferred to a sterile microcentrifuge tube.

#### Real-time PCR amplification

For the 14 salivary bacteria species, real-time PCR (qPCR) amplification was performed on the mask, UFR, and SFR samples using primers specific for each. The amounts of total bacteria were quantified using 16S ribosomal RNA (rRNA) primers from each bacterium. Primers were same as those used in our previous study (19). To obtain the total bacterial DNA copy number, a conservative bacteria 16s RNA primer probe was used to measure the total DNA copy number (Table 1).

**Table 1:** Bacteria 16s RNA primer probe for quantifying total bacteria.

No	Name	Sequence	Bp
1	Forward	CTCAAAKGAATTGACGGGG	19
2	Reverse	GTCATCCMMACCTTCCTC	18
3	Probe	5′Cy5-CATGGYTGTCGTCAGCTCGTG-3′BHQ2	21

A reaction mixture consisting of 5 μl of DNA template, 2.5 pM forward and reverse primers, and 10 μl of 2X master mix (GeNet Bio, Daejeon, Korea) was used. Twenty microliters of the total reaction mixture were pipetted and used for qPCR. The qPCR process consisted of pre-denaturation at 95°C for 10 min, 45 cycles of denaturation at 95°C for 15 s, and annealing and extension at 60°C for 1 min (20). In this process, plasmid DNA synthesized from each bacterium and DNase/RNase-free water were used as positive and negative controls, respectively.

#### Calculation of DNA copy number of oral bacteria

UFR and SFR samples were prepared by mixing 2 ml of saliva and 2 ml of sample stock solution. The mask sample was prepared by placing the mask in 20 ml of sample preservation solution. For all three types of samples (UFR, SFR, and mask), 500 µl of the sample preservation solution was used for DNA extraction, and the final 100 µl was eluted. After performing real-time PCR using 5 µl (out of 100 µl) of the total solution, DNA was quantified using the standard curve method (quantitative value for 5 µl used for analysis). Finally, we checked the copy number in 1 ml of saliva and 20 ml of preservation solution. Components of the preservative solution included tris-HCl, urea, sodium acetate, sodium dodecyl sulfate, ethylene-diamine-tetra acetic acid, sodium ascorbate, and ethanol.

#### α-Diversity based on the shannon diversity index

α-Diversity was calculated using the Shannon diversity index and bacterial richness was measured as the total number of bacterial DNA copies. The α-diversity levels of microbial profiles were compared using Shannon index calculations using the following formula (21):

H=–∑pi×ln(pi)

where pi = n/N is the relative abundance, n is the number of given species, N is the total number of bacterial species in a community, H is the Shannon diversity index, pi is the proportion of cells of the i^th^ species in the whole community, pi = n/N, where n is the number of cells of a given taxon/species, and N is the total number of bacterial cells in a community. The minimum value (zero) of the Shannon diversity index indicates no diversity; therefore, greater the value, higher the diversity. In real-world ecological data, the Shannon diversity index typically ranges from 1.5 to 3.5, rarely reaches 4.5 (22).

### Statistical analysis

The absolute and percentage distributions, means and standard deviations of all nominal and categorical variables were obtained, and descriptive data analysis was performed. Various statistical methods were used for data analysis. First, oral bacteria in the three groups were investigated. An analysis of variance (ANOVA) was used to investigate the differences in mean values related to the oral microbiome among the three groups. The results of participants with and without xerostomia were compared using the Mann–Whitney U test. To analyze the distribution of discontinuous data, we used the *χ*^2^ test, Fisher's exact test, and the Bonferroni test for equality of proportions. Spearman's correlation analysis was used to determine the correlations between variables, and the correlation coefficient (r) was closer to the absolute value of 1, indicating a stronger correlation (23). In the multiple linear regression model, the dependent variable of the total number of bacteria was considered continuous, and each bacterium was considered an independent variable. The estimated beta (β), standard error (SE), and 95% confidence interval (CI) were calculated using linear regression analyses with age adjustment. Statistical significance was set at *p* < 0.05. Data were analyzed using IBM SPSS Statistics for Windows (version 24.0; IBM Corp., Armonk, NY, USA). Shannon's diversity index was calculated in R (Version 4.0.2; R Foundation for Statistical Computing, Vienna, Austria).

## Results

Of the 55 subjects, 14 (25.5%) had xerostomia, and consequently, UFR was significantly lower in the xerostomia group than in the non-xerostomia group (0.92 ± 0.13 vs. 1.10 ± 0.37 ml/min, *p* < 0.05). However, there were no statistically significant differences in age, gender ratio, salivary pH, saliva buffer capacity, and mask-wearing time between the xerostomia and non-xerostomia groups (Table 2). The mean values of salivary pH (7.16 ± 0.47) and buffer capacity (10.01 ± 0.95) for all participants were within the normal range.

**Table 2:** Demographics, clinical characteristics, and volatile sulfite compounds levels of participants.

	Total (*n* = 55)	Non-xerostomia (*n* = 41)	Xerostomia (*n* = 14)	Non-xerostomia vs. Xerostomia
	Mean ± SD or *n* (%)	Mean ± SD or *n* (%)	Mean ± SD or *n* (%)	*p*-value
Epidemiology				
Age (years)^a^	38.18 ± 12.49	38.78 ± 12.42	36.43 ± 12.99	0.560
Sex (female)^b^	45 (81.8%)	33 (80.5%)	12 (85.7%)	1.000
Saliva				
UFR (ml/min)^a^	1.06 ± 0.33	1.10 ± 0.37	0.92 ± 0.13	**0**.048*
SFR (ml/min)^a^	1.41 ± 0.42	1.43 ± 0.44	1.38 ± 0.34	0.573
Salivary pH^a^	7.16 ± 0.47	7.15 ± 0.51	7.17 ± 0.34	0.868
Buffer capacity^a^	10.01 ± 0.95	10.00 ± 1.04	10.07 ± 0.62	0.743
Mask wearing duration (hours)^a^	5.91 ± 2.95	5.76 ± 2.89	6.36 ± 3.20	0.541

Results were obtained using.

^a^ Mann–Whitney *U* test.

^b^ Chi-square test (two-sided).

A *p*-value < 0.05 (*p < 0.05) was considered significant. Significant results are shown in bold text.

### DNA copy numbers of each bacterium and “red complex”

**Figure 1:**
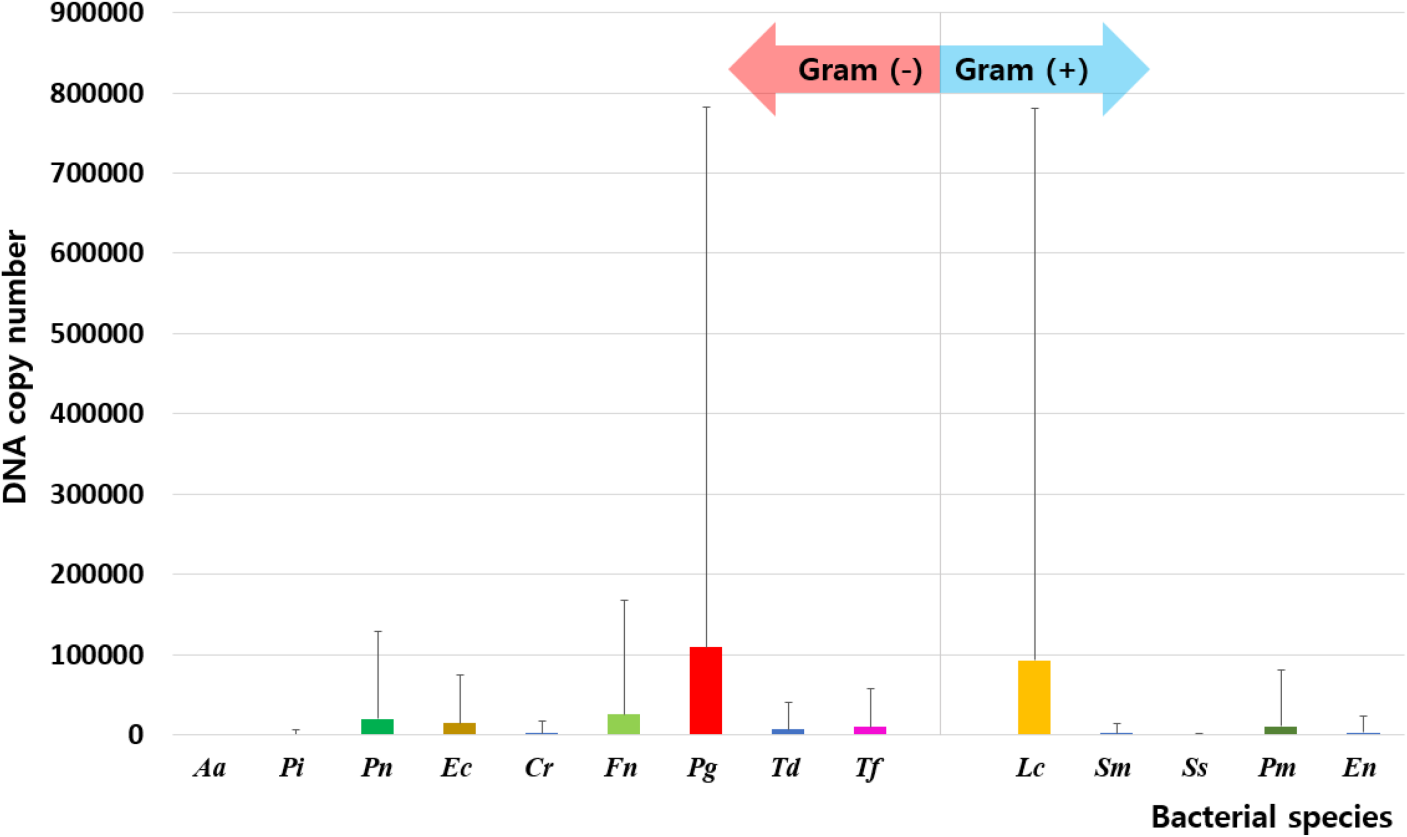
Distribution of oral bacteria on the inner surface of the mask. Aa, aggregatibacter actinomycetemcomitans; Pi, prevotella intermedia; Pn, prevotella nigrescens; Ec, eikenella corrodens; Cr, campylobacter rectus; Fn, fusobacterium nucleatum; Pg, porphyromonas gingivalis; Td, treponema denticola; Tf, tannerella forsythia; Lc, lactobacillus casei; Sm, streptococcus mutans; Ss, streptococcus sobrinus; Pm, parvimonas micra; En, eubacterium nodatum.

Of the 14 species investigated in this study, 9 [*A. actinomycetemcomitans*, *P. intermedia*, *P. nigrescens*, *E. corrodens*, *C. rectus*, *F. nucleatum*, *P. gingivalis*, *T. denticola*, and *T. forsythia*] were Gram-negative, and 5 [*L. casei*, *S. mutans*, *S. sobrinus*, *P. micra*, and *E. nodatum*] were Gram-positive. Total DNA isolated from oral bacteria was significantly higher in UFR (43,214,244.45 ± 86,900,936.03) and SFR (41,015,254.15 ± 109,947,416.90) than in mask (299,449.14 ± 1,356,728.10, *p* < 0.001). On the inner surface of the mask, Gram-negative and Gram-positive bacteria had abundances in the order: *P. gingivalis > F. nucleatum > P. nigrescens > E. corrodens > T. forsythia *> *T. denticola* and *L. casei > P. micra *> *E. nodatum*, respectively (Figure 1). The DNA copy number of total bacterial and Gram-negative species was significantly higher in UFR and SFR than in mask, whereas no significant difference between UFR, SFR, and mask in the amount of Gram-positive species was observed. The “red complex” is an aggregate of three oral bacterial species (*P. gingivalis*, *T. forsythia*, and *T. denticola*) responsible for severe clinical manifestations of periodontal disease (24). The abundance of red complexes was significantly higher in UFR than in mask.

Among Gram-negative bacteria, the number of *P. intermedia, E. corrodens, P. gingivalis*, and *T. forsythia* was significantly higher in UFR than in mask, but there were no significant differences between SFR and mask. The number of *F. nucleatum*, *P. nigrescens, C. rectus, and T. denticola* was significantly higher in both UFR and SFR than in mask samples (all *p* < 0.05). For Gram-positive bacteria, the amount of *P. micra* was significantly higher in UFR than in mask (Table 3).

**Table 3:** Comparison of DNA copies of each bacterium, red complex species, and Shannon's diversity index of 14 bacterial species.

	Mask	UFR	SFR	*p*-value	ANOVA post-hoc
	Mean ± SD	Mean ± SD	Mean ± SD		
Gram (−)					
*Aa*	0.0 ± 0.0	84,755.60 ± 578,052.88	134,356.69 ± 540,986.39	0.299	
*Pi*	1,009.07 ± 4,756.38	3,252,431.72 ± 10,798,693.98	2,621,023.11 ± 6,569,457.88	**0**.049*	UFR > Mask
*Pn*	20,065.93 ± 108,399.48	7,788,593.60 ± 13,760,420.11	5,776,950.63 ± 9,490,677.27	**0**.000***	UFR, SFR > Mask
*Ec*	14,441.06 ± 60,449.39	2,922,048.32 ± 7,027,864.73	1,559,684.78 ± 2,837,115.07	**0**.003**	UFR > Mask
*Cr*	2,420.45 ± 14,756.47	806,903.23 ± 2,686,562.33	321,813.35 ± 1,056,329.93	**0**.041*	UFR, SFR > Mask
*Fn*	25,462.62 ± 141,779.44	10,531,300.90 ± 17,370,158.89	7,175,748.41 ± 10,887,153.63	**0**.000***	UFR, SFR > Mask
*Pg*	109,153.86 ± 673,306.71	13,088,432.59 ± 37,707,571.96	7,136,010.12 ± 19,615,461.35	**0**.023*	UFR > Mask
*Td*	6,748.89 ± 33,845.44	477,025.28 ± 989,482.40	372,568.44 ± 738,112.41	**0**.002**	UFR, SFR > Mask
*Tf*	10,335.07 ± 47,508.19	1,749,732.47 ± 3,972,943.97	1,055,919.86 ± 2,316,593.77	**0**.003**	UFR > Mask
Gram (+)					
*Lc*	93,236.38 ± 687,696.80	14,219.64 ± 95,547.16	13,235,096.48 ± 98,117,235.79	0.373	
*Sm*	2,256.01 ± 12,020.02	140,262.82 ± 726,608.70	171,988.60 ± 619,753.35	0.232	
*Ss*	310.76 ± 1,671.51	1,413.51 ± 7,256.32	10,774.20 ± 72,600.66	0.361	
*Pm*	10,823.97 ± 70,561.99	1,844,955.69 ± 5,906,186.05	1,176,769.25 ± 3,274,469.32	**0**.047*	UFR > Mask
*En*	3,185.06 ± 20,219.21	512,169.07 ± 1,918,624.16	266,550.33 ± 758,378.21	0.084	
Total bacteria	299,449.14 ± 1,356,728.10	43,214,244.45 ± 86,900,936.03	41,015,254.15 ± 109,947,416.90	**0**.000***	UFR, SFR > Mask
Gram (−) total	164,174.34 ± 935,102.95	30,169,922.80 ± 66,728,696.32	18,978,326.97 ± 35,550,304.76	**0**.002**	UFR, SFR > Mask
Gram (+) total	135,274.81 ± 726,509.83	13,044,321.63 ± 22,908,382.41	22,036,927.28 ± 99,405,273.61	0.15	
Red complex	126,237.82 ± 753,796.99	15,315,190.34 ± 41,966,894.16	8,564,498.42 ± 22,077,308.09	**0**.016*	UFR > Mask
Diversity index					
Shannon's diversity index	1.26 ± 1.51	2.64 ± 0.78	2.66 ± 0.76	**0**.000***	UFR, SFR > Mask

Results were obtained using ANOVA and *post hoc* analysis. Differences between sample types were considered significant at *p*-value < 0.05. Significant differences are shown in bold.

Aa, aggregatibacter actinomycetemcomitans; Pi, prevotella intermedia; Pn, prevotella nigrescens; Lc, lactobacillus casei; Fn, fusobacterium nucleatum; Sm, streptococcus mutans; Ss, streptococcus sobrinus; Td, treponema denticola; Pg, porphyromonas gingivalis; Tf, tannerella forsythia; Ec, eikenella corrodens; Pm, parvimonas micra; Cr, campylobacter rectus; En, eubacterium nodatum.

Mask, DNA copy number of bacteria inside the mask; UFR, bacterial DNA copy number under unstimulated salivary flow; SFR, bacterial DNA copy number under stimulated salivary flow; Gram (−), Gram-negative species (*Aa, Pi, Pn, Ec, Cr, Fn, Pg, Td*, and *Tf*); Gram (+), Gram-positive species (*Lc, Sm, Ss, Pm*, and *En*); Red complex, pathogenic consortium of periodontitis (*Pg, Td*, and *Tf*).

* *p* < 0.05.

** *p* < 0.01.

*** *p* < 0.001.

Interestingly, the DNA copy number of each of the 14 bacteria, total bacterial amount, and Shannon's diversity index did not differ between participants with or without xerostomia, and the presence or absence of xerostomia did not significantly affect the UFR, SFR, or mask samples.

### Shannon's diversity index

**Figure 2:**
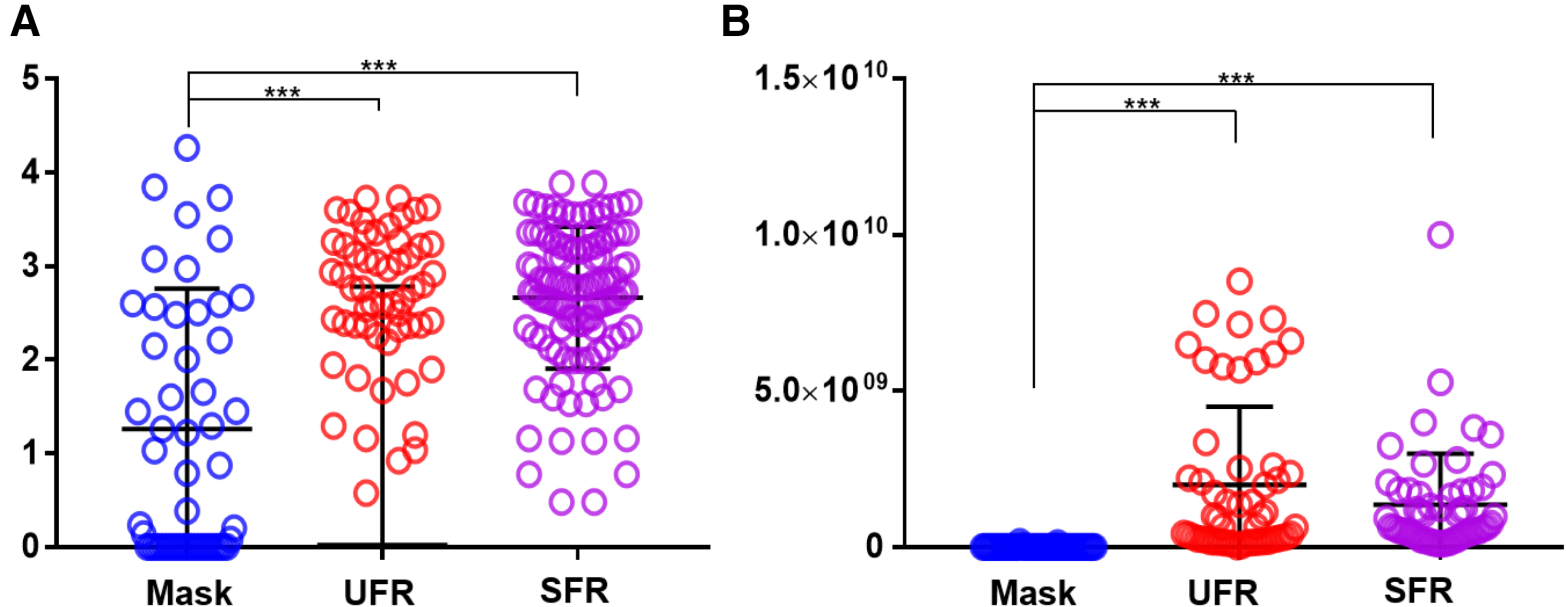
Comparison of Shannon's diversity index and amounts of total oral bacteria in mask, UFR, and SFR samples. (**A**) Shannon's diversity index, (**B**) Total bacterial DNA copy number. The results were obtained using ANOVA. Mask, inside the mask; UFR, under unstimulated salivary flow rate; SFR, under stimulated salivary flow. Differences as derived from ANOVA modeling were considered significant at *p* < 0.05 (***p < 0.001).

**Figure 3:**
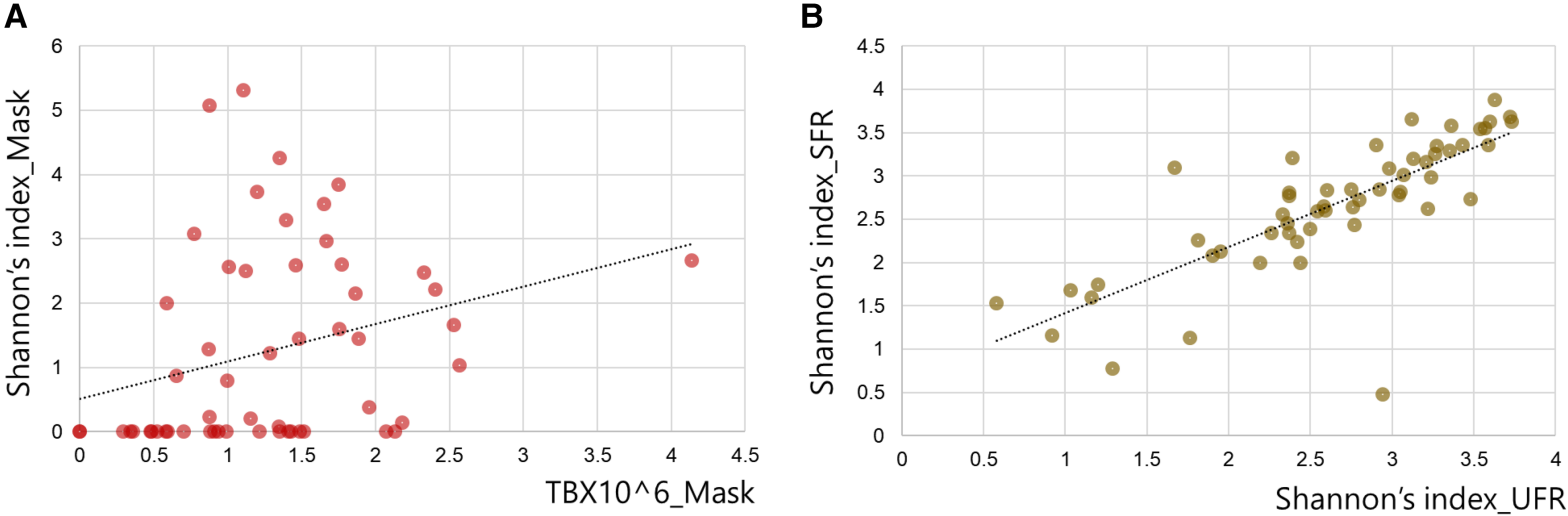
Correlation between Shannon's diversity index of saliva and the inner mask. (**A**) Correlation between Shannon's diversity index and total bacterial DNA copy number (TB) of the inner mask. (**B**) Correlation between Shannon's diversity index of SFR and the value of UFR.

Shannon's diversity index was significantly higher in UFR and SFR than in mask (2.64 ± 0.78, 2.66 ± 0.76, and 1.26 ± 1.51, respectively; *p* < 0.001) (Figure 2). The total bacterial DNA copy number collected from the inner surface of the mask was significantly positively correlated with Shannon's diversity index of the mask (*r* = 0.510, *p* < 0.01). Although Shannon's diversity indices of UFR and SFR were significantly positively correlated (*r* = 0.828, *p* < 0.001), no significant relationship was found with Shannon's diversity index of the mask (Figure 3). In other words, the diversity of oral bacteria inside the mask was independent from that of saliva.

### Factors affecting oral bacteria on the inner surface of the mask

With increasing age of participants, *P. intermedia* (*r* = 0.185), *C. rectus* (*r* = 0.204), *P. gingivalis* (*r* = 0.021), *T. denticola* (*r* = 0.185), and *F. nucleatum* (*r* = 0.113) in mask samples increased significantly (all *p* < 0.05). Total bacterial DNA copy number also increased with age (*r* = 0.162, *p* < 0.05). A decrease in the UFR, significantly increased the amount of *S. mutans* (*r* = −0.268, *p *< 0.05). Whereas, a decrease in the SFR significantly increased the *P. intermedia* (*r* = −0.301), *L. casei* (*r* = −0.270), and *P. micra* (*r* = −0.270) (all *p* < 0.05). In this study, salivary pH, salivary buffer capacity, mask wearing time, and xerostomia were not correlated with the amounts of bacteria, total bacteria, or the red complex, including *P. gingivalis*, *T. denticola*, and *T. forsythia* (Table 4).

**Table 4:** Correlation between clinical indicators and oral bacteria of inner surface of the mask.

r	*Aa* Mask	*Pi* Mask	*Pn* Mask	*Ec* Mask	*Cr* Mask	*Pg* Mask	*Td* Mask	*Tf* Mask	*Lc* Mask	*Fn* Mask	*Sm* Mask	*Ss* Mask	*Pm* Mask	*En* Mask	TB Mask	Red complex	Shannon's diversity
Age		**0**.**185**	−0.017	0.206	**0**.**204**	**0**.**021**	**0**.**081**	−0.251	0.234	**0**.**113**	−0.072	0.105	−**0**.**098**	−0.164	**0**.**162**	−**0**.**011**	0.233
UFR		−0.144	−0.042	0.017	−0.011	−0.023	−0.130	0.019	−0.155	0.013	−**0**.**268**	−0.022	0.063	0.234	−0.162	−0.077	−0.055
SFR		−**0**.**301**	−0.081	−0.070	−0.265	−0.140	−0.034	−0.163	−**0**.**270**	−0.230	−0.070	−0.127	−**0**.**270**	−0.091	−0.217	−0.114	−0.028
Salivary pH		0.027	−0.012	−0.015	−0.042	−0.037	−0.039	−0.132	−0.055	0.018	0.060	−0.016	−0.128	−0.068	−0.088	−0.133	−0.032
Buffer capacity		−0.116	0.090	0.128	0.090	0.084	0.216	0.094	−0.019	−0.176	0.121	0.135	0.050	0.190	0.102	0.013	0.055
Mask wearing time		0.153	0.080	0.064	0.005	0.057	0.127	−0.093	0.194	0.064	0.012	−0.097	−0.041	0.028	0.087	0.013	0.035
Xerostomia		−0.185	−0.017	0.206	−0.204	−0.021	0.081	−0.251	0.234	−0.113	−0.072	0.105	−0.098	−0.164	−0.058	−0.011	0.098

Results were obtained via Spearman's correlation analysis. *P*-value was considered as significant when *p*-value < 0.05 (*p < 0.05). Significant results shown in bold text. r, correlation coefficient; *Bacterium name* Mask, bacterial abundance in mask samples; TB, DNA copy number of collected total bacteria; Red complex, DNA copy number of pathogenic consortium of periodontitis (*Pg*, *Td*, and *Tf*); Shannon's diversity, Shannon's diversity index of collected total bacteria; UFR, unstimulated whole saliva; SFR, stimulated whole saliva.

Aa, aggregatibacter actinomycetemcomitans; Pi, prevotella intermedia; Pn, prevotella nigrescens; Lc, lactobacillus casei; Fn, fusobacterium nucleatum; Sm, streptococcus mutans; Ss, streptococcus sobrinus; Td, treponema denticola; Pg, porphyromonas gingivalis; Tf, tannerella forsythia; Ec, eikenella corrodens; Pm, parvimonas micra; Cr, campylobacter rectus; En, eubacterium nodatum.

### Multiple linear regression analysis of oral bacterial amounts

In the multiple linear regression analysis, the total number of bacteria inside the mask was used as a dependent variable, and the bacterial species present on the mask were used as independent variables. The main bacteria that increased the total bacterial abundance in mask samples were *F. nucleatum* (β = 1,292.72, 95% CI = 947.34, 1,638.11), *P. gingivalis* (β = 133.82, 95% CI = 81.16, 186.48), and *E. corrodens* (β = 84.80, 95% CI = 10.85, 158.75) (*R* = 0.996, adjusted *R*^2^ = 0.989). In the UFR and SFR samples, *F. nucleatum* majorly contributed to the increase in the amount of total bacterial DNA, but the contribution and significance of other bacterial species were different from those of the mask samples (UFR: *R* = 0.956, adjusted *R*^2^ = 0.884, and SFR: *R* = 0.967, adjusted *R*^2^ = 0.913) (Table 5).

**Table 5:** Linear regression analysis regarding total amount of oral bacteria as independent variable.

Dependent variable	Total bacteria_Mask					Total bacteria_UFR					Total bacteria_SFR				
	β	SE	*p*-value	95% CI Lower	95% CI Upper	β	SE	*p*-value	95% CI Lower	95% CI Upper	β	SE	*p*-value	95% CI Lower	95% CI Upper
Gram (−)															
*Aa*						190.436	363.822	.604	−544.876	925.748	15.802	154.631	.919	−296.719	328.324
***Pi***	**−745**.**70**	120.54	**0**.**00**	−989.14	−502.26	29.19	34.76	0.41	−41.05	99.44	40.89	45.90	0.38	−51.88	133.66
***Pn***	−147.95	75.41	0.06	−300.25	4.35	**72**.**48**	29.55	**0**.**02**	12.75	132.21	−0.40	19.77	0.98	−40.35	39.56
***Ec***	**84**.**80**	36.62	**0**.**03**	10.85	158.75	20.54	36.61	0.58	−53.46	94.53	36.02	39.58	0.37	−43.98	116.01
*Cr*	681.98	1,013.72	0.50	−1,365.27	2,729.24	60.55	177.14	0.73	−297.46	418.56	98.27	240.92	0.69	−388.64	585.18
***Fn***	**1,292**.**72**	171.02	**0**.**00**	947.34	1,638.11	**168**.**89**	13.73	**0**.**00**	141.13	196.64	**138**.**64**	20.18	**0**.**00**	97.85	179.42
***Pg***	**133**.**82**	26.07	**0**.**00**	81.16	186.48	**56**.**57**	20.56	**0**.**01**	15.01	98.13	**−66**.**88**	31.13	**0**.**04**	−129.79	−3.98
*Td*	114.71	128.68	0.38	−145.16	374.57	−419.27	316.79	0.19	−1,059.52	220.97	−336.28	219.68	0.13	−780.26	107.70
***Tf***	146.46	108.63	0.19	−72.94	365.85	−239.98	186.53	0.21	−616.97	137.00	**373**.**41**	174.11	**0**.**04**	21.53	725.29
Gram (+)															
*Lc*	0.33	0.67	0.63	−1.03	1.69	−699.21	1,231.24	0.57	−3,187.64	1,789.21	−0.70	0.71	0.33	−2.14	0.74
***Sm***	10.00	281.40	0.97	−558.30	578.31	240.32	171.26	0.17	−105.81	586.45	**367**.**37**	110.64	**0**.**00**	143.76	590.99
*Ss*	−91.44	271.14	0.74	−639.02	456.15	15,661.22	13,553.27	0.41	−22,621.20	53,943.64	−1,664.79	5,381.93	0.76	−12,542.08	9,212.51
***Pm***	**−563**.**08**	113.83	**0**.**00**	−792.97	−333.20	**−546**.**16**	81.58	**0**.**00**	−711.04	−381.27	158.19	138.54	0.26	−121.80	438.18
***En***	**−1,991**.**18**	386.89	**0**.**00**	−2,772.51	−1,209.85	−71.09	159.11	0.66	−392.66	250.48	−351.32	348.03	0.32	−1,054.72	352.07

Results were obtained via linear regression analysis. Statistical significance was set as significant when *p*-value < 0.05. The significant results are shown in bold. β, estimated beta; SE, standard error; CI, confidence interval; Total bacteria_Mask, DNA copy number of total bacteria inside the mask; Total bacteria_UFR, Total bacterial DNA copy number under unstimulated salivary flow rate measurement conditions; Total bacteria_UFR, Total bacterial DNA copy number under stimulated salivary flow rate measurement conditions. Gram (−), Gram-negative bacterial species, Gram (+), Gram-positive bacterial species.

Aa, aggregatibacter actinomycetemcomitans; Pi, prevotella intermedia; Pn, prevotella nigrescens; Lc, lactobacillus casei; Fn, fusobacterium nucleatum; Sm, streptococcus mutans; Ss, streptococcus sobrinus; Td, treponema denticola; Pg, porphyromonas gingivalis; Tf, tannerella forsythia; Ec, eikenella corrodens; Pm, parvimonas micra; Cr, campylobacter rectus; En, eubacterium nodatum.

## Discussion

Droplets and aerosols produced by both non-violent and violent exhalation from people infected with SARS-CoV-2 can lead to the airborne transmission of COVID-19. Wearing a mask is an effective and economical way to prevent transmission during the COVID-19 pandemic, where there is a shortage of efficient treatments (25). In this study, the bacterial profile of the inner surface of the masks was investigated in connection with the salivary microbiome, focusing on nine Gram-negative [*A. actinomycetemcomitans, P. intermedia, P. nigrescens, E. corrodens, C. rectus, F. nucleatum, P. gingivalis, T. denticola*, and *T. forsythia*] and five Gram-positive bacterial taxa [*L. casei, S. mutans, S. sobrinus, P. micra*, and *E. nodatum*]. Both unstimulated and stimulated saliva were investigated. We found a significant correlation between salivary bacteria and oral bacteria on the inner surface of the mask. The amount and diversity of oral bacteria on the mask were lower than those in the UFR and SFR samples, yet the mask samples still had a unique oral microbiome profile.

Since masks were donned in a sterilized state, the oral bacteria on the inner surface can be inferred to be derived from the oral saliva. However, the microbiome profile of the mask was independent of that of saliva. On the inner surface of the mask, Gram-negative and Gram-positive bacteria had abundances in the following order: *P. gingivalis > F. nucleatum > P. nigrescens > E. corrodens > T. forsythia > T. denticola* and *L. casei > P. micra *> *E. nodatum*, respectively*.* Oral bacteria have the ability to adhere to various surfaces of the oral ecosystem, allowing them to integrate into the resident microbiome, which favors their growth and survival (26). Notably, different bacterial species in the oral cavity may have different adhesion and proliferation abilities on the inner surface of the mask. *P. gingivalis*, an anaerobic Gram-negative bacterium that is a keystone oral pathogen in periodontitis (27), was detected in 25% of periodontally healthy participants (28). Red complexes, including *P. gingivalis*, *T. forsythia*, and *T. denticola*, prevent the oral bacterial layer from being easily removed and alter the composition of the surrounding oral microbiome (29), which can worsen periodontal conditions. On the inner surface of the mask, *P. gingivalis* was the most abundant Gram-negative species. According to our previous study, an increase in *P. gingivalis* inside the mask was also related to an increase in volatile sulfide compound level, which is a major factor in halitosis, along with other salivary bacteria such as *T. denticola*, *T. forsythia, P. intermedia*, and *P. nigrescens* (19). Interestingly, the presence or absence of xerostomia had no impact on the profile of the oral microbiome on the inside of the mask. A recent study on the mask microbiome conducted on dental students reported that the severity of xerostomia was not significantly related to mask wearing time (16). Lin et al. reported that *P. gingivalis* and *F. nucleatum* were clearly associated with xerostomia after radioiodine therapy (30). However, the occurrence of xerostomia and changes in the red complex following mask wearing in the general population require further investigation. The abundances of red complex were significantly lower in the mask than in UFR but not significantly different from that in SFR. *L. casei*, a Gram-positive, non-motile bacterium that is widely used in probiotics, has been reported to alleviate multiple diseases. In particular, *L. casei* inhibits *P. gingivalis* (31). Further research is needed to elucidate bacterial interactions inside the mask.

Some salivary microbiomes adhere and proliferate relatively easily on the inner surface of the masks. The total DNA copy number of oral bacteria was significantly higher in UFR and SFR than in mask, with average value approximately 130 times higher. Shannon's diversity index is a quantitative indicator of the number and type of bacterial signatures present in a sample while taking into accounts the uniformity of the distribution of these bacteria (32). Shannon's index of UFR and SFR had a significant strong positive correlation with each other (*r* = 0.828, *p* < 0.001), but not with the mask samples. Shannon's index usually decreases in patients suffering from diseases and its decrease or increase can have complex causes (21). Shannon's diversity index of mask was 1.26 ± 1.51, which was significantly lower than that of UFR (2.64 ± 0.78) and SFR (2.66 ± 0.76). In a previous study that investigated Shannon's diversity index in various habitats of the oral cavity, the median values of saliva, buccal mucosa, and tongue were 2.308, 1.413, and 2.095, respectively, which was higher than that of the tooth surface, whereas it was 2.343 times lower than that of the subgingival plaque (33). The reason for the high diversity in bacterial-suspended saliva and subgingival plaques, which are protected by biofilms and periodontal pocket walls, is unclear. In fact, diverse oral bacterial species are associated with healthy gingiva, including Gram-positive cocci, a small number of Gram-positive bacilli, and a very small number of Gram-negative cocci (34). As the present study is the first to examine the oral microbiome of the inner surface of masks, no comparable previous results are available. The specific area of the oral cavity that reflects the detected composition should be investigated.

Although information on mask-related oral microbiome changes in the general population is lacking, it may be expected that bacterial composition is affected by age and salivation rate. In this study, a weak positive correlation was found between increasing age and abundance of the Gram-negative species, including *P. intermedia, C. rectus, P. gingivalis,* and *T. denticola*, all of which are important markers that play a pathogenic role in destructive periodontal disease in adults (35–37). Periodontitis can occur at any age, with a general prevalence of approximately 14% that increases with age (38). Therefore, an increase in causative bacteria can be expected with progressive aging. Interestingly, an increase in salivation rate decreased the abundance of some bacteria in the mask, such as *S. mutans*, an anaerobic Gram-positive coccus that is a significant contributor to dental caries (39). A similar decrease was observed for *P. intermedia, L. casei,* and *P. micra*. Since saliva promotes enzymatic degradation of bacterial cell walls via lysozyme action and iron sequestration by lactoferrin (40), oral pathogens such as *S. mutans* and *P. intermedia* can be expected to decrease when salivary flow increases; however, no previous study has yet illustrated this. Moreover, no studies have reported a relationship between beneficial *L. casei* (a representative probiotic) and salivary flow rate.

Oral bacteria on the inside of these masks have the potential to cause skin problems. Park et al. recently investigated skin changes caused by mask-wearing and changes over time during the COVID-19 pandemic and found that skin temperature, redness, hydration, and sebum secretion were changed significantly after 1 and 6 h of wearing a mask (41). In addition, these parameters differed significantly between mask-wearing and non-mask-wearing areas (41). Since sterile KF94 masks were initially used in the present clinical trial, the bacteria later detected on the inside of the mask would be expected to be mainly derived from the oral cavity or the skin around the mouth (42). Bacteria proliferating on the inside of the mask may adversely affect the skin area covered by the mask. Approximately 90% of skin infections are caused by *Staphylococcus aureus* and *Streptococcus pyogenes*, and some anaerobic bacteria such as *Prevotella* species and *Bacteroides* are also considered important causes of skin infections (43, 44). *Porphyromonas gingivalis* is the dominant pathogen of dysbiosis and periodontitis of the oral cavity (45). *P. gingivalis* is aerotolerant and can proliferate in microenvironments with low oxygen (46). In the present study, the dominant species inside the mask was *P. gingivalis.* Although *Porphyromonas* species are more commonly associated with oral infections, the occurrence of skin problems caused by *P. gingivalis* has recently been reported (47). None of the participants in our study suffered from a clinically noticeable skin condition, and no scientific investigations or surveys were conducted on skin problems. Therefore, additional research is needed to determine which skin problems may be caused by these oral pathogens in the environment created by masks, and which key species may be involved.

This study aimed to investigate and report the diversity of the oral microbiome, the abundance of each bacterium on the inner surface of the mask, and the factors that influence bacterial diversity. Furthermore, we compared these trends with those of whole saliva. Masks have been used since the 17th century to treat European epidemics (48), and the masks have helped mankind a lot in preventing the spread of coronavirus during the COVID-19 pandemic. Based on this study, it is advisable to keep the inner surface of the mask as clean as possible to reduce the potential bacterial side effects of mask-wearing. This study also provides an explanation for the mechanism of dermal issues and the rationale for frequent mask replacement. A limitation of our study is that female participants were outnumbered by male participants because participants were recruited through in-hospital advertisements, and children, adolescents, and older people were not included, which may cause a bias in the results and conclusions depending on the age or gender of the participants. Since the study design targeted consecutive volunteers who expressed their intention to participate during the study period, the number of participants with and without xerostomia was disproportionate. Furthermore, only 14 representative types of bacteria constituting the oral microbiome have been identified. To clearly interpret the results of the mask-wearing, additional large-scale studies based on next-generation sequencing and multi-omics to analyze whole bacteria, fungi, and viruses are needed.

## Data Availability

The raw data supporting the conclusions of this article will be made available by the authors, without undue reservation.
